# Microleakage along the implant–abutment interface: a systematic review and meta-analysis of in vitro studies

**DOI:** 10.1186/s40729-023-00494-y

**Published:** 2023-09-21

**Authors:** Zhen Mao, Florian Beuer, Daomin Wu, Qiuyan Zhu, Jamila Yassine, Andreas Schwitalla, Franziska Schmidt

**Affiliations:** 1 Charité–Universitaetsmedizin Berlin, Corporate Member of Freie Universität Berlin and Humboldt- Universitaet zu Berlin, Dental Materials and Biomaterial Research, Department of Prosthodontics, Geriatric Dentistry and Craniomandibular Disorders, Aßmannshauser Str. 4-6, 14197, Berlin, Germany; 2 Department of Oral Implantology, The Affiliated Stomatology Hospital of Kunming Medical University, Block C, No. 1088, Haiyuan Middle Road, High-Tech Zone, Kunming, Yunnan China

**Keywords:** Bacterial leakage, Implant–abutment interface, Sealing capability, Systematic review, Meta-analysis

## Abstract

**Purpose:**

This systematic review aimed to evaluate the incidence of microleakage events (IME) and to identify the potential factors influencing the sealing ability of the implant–abutment interface (IAI) under in vitro investigation.

**Material and methods:**

An electronic search of MEDLINE (PubMed), EMBASE, and Web of Science databases, combined with a manual literature search was conducted up to September 2022. In vitro studies that reported the degree of microleakage at IAI under dynamic loading conditions were included. A meta-analysis was performed to calculate the mean values of the incidence of microleakage events. Subgroup analysis and meta-regression were conducted to further investigate the effect of different variables.

**Results:**

675 studies were identified following the search process and 17 in vitro studies were selected according to the eligibility criteria. The weighted mean incidence of microleakage events was 47% (95% confidence interval: [0.33, 0.60]), indicating that contamination was observed in nearly half of the samples. Concerning possible factors that may influence microleakage (e.g., loading condition, assessment method, implant–abutment connection design, types of abutment material, the use of sealing agents), loading condition (*p* = 0.016) was the only variable that significantly influenced IME in the meta-regression analysis.

**Conclusions:**

The results demonstrated that dynamic loading significantly increases the potential of bacterial penetration at the implant–abutment junction. The results should be interpreted carefully due to the data heterogeneity and further well-conducted in vitro studies with homogeneous samples are needed to standardize the methodologies.

**Graphical Abstract:**

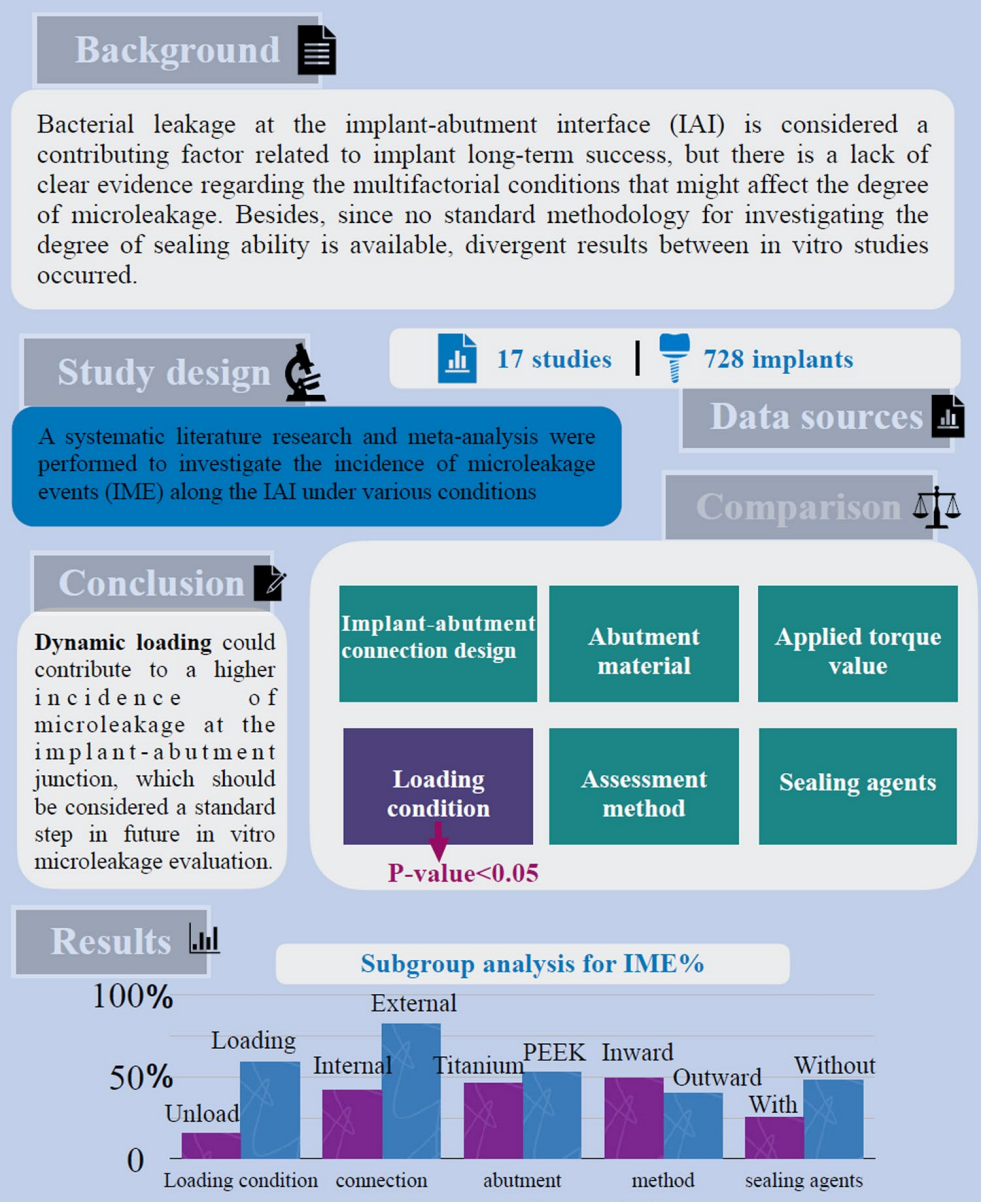

## Background

Two-piece dental implant systems consisting of an endosseous implant and a prosthetic abutment have been increasingly used and are considered a suitable treatment option for teeth replacement and fixation support. However, the presence of a microgap at the implant–abutment interface may lead to bacterial leakage, which can contribute to peri-implantitis [1]. Thus, improving the sealing ability of the implant–abutment interface (IAI) against bacterial colonization might be a factor for long-term success. Multifactorial conditions might affect the degree of microleakage along the IAI, including but not limited to the accuracy of the implant–abutment union, final torque force, microbial species, the use of sealing material, and the micromovements of the different components during the masticatory cycles.

Furthermore, the precision of fit between components is partly based on the geometry of IAI and it has been argued that the internal design, especially for conical connections is more efficient than external connections in preventing bacterial penetration [2]. On the other hand, Schmitt et al. argued that almost no IAI connection type can completely protect implants from bacterial contamination [3]. Moreover, it has been reported that the use of different abutment materials [e.g., titanium, polyetheretherketone (PEEK)] and the placement of sealing agents such as silicon sealant may also influence the degree of microleakage along the IAI [4–6]. However, there is no review quantitatively and systematically investigating the incidence of microleakage events at the IAI with various connection designs and related factors.

In addition, several methodologies have been developed to investigate the microleakage along the IAI. For example, most related studies analyzed the bacterial invasion from the outside to the inner parts of the implant (named here as “inward method”) by assembling samples inside a marker solution and testing for penetration from the inner portion of the implant body afterward [7–11]. Others inoculate the implant body with marker solution before the abutment connection placement and test the leakage of marker on the outside of the implants (named here as “outward method”) [12–14]. Likewise, microscopy, X-ray computed tomography, and bacterial DNA analysis have been employed in vitro to investigate bacterial leakage [10, 15]. In addition, compared with testing under dynamic loading, IAI shows better sealing ability in static or unloaded condition, which may be due to the micromovement at the IAI which causes a pumping effect [1, 16, 17]. Conversely, Mishra et al. proposed that a conical internal connection showed better performance under dynamic loading. A major reason for this phenomenon was that the loading force may reduce the size of micro gaps to limit the penetration of the microbes [18]. Indeed, the divergent results may be explained by the lack of standard in vitro methodologies and the heterogeneity among in vitro experiments. Therefore, the purposes of the present review were as follows:

1. To investigate the sealing ability along the IAI and to identify the factors influencing the incidence of microleakage events.
2. To evaluate the effect of methodological aspects of in vitro studies on the leakage outcomes along the IAI.

## Methods

### Protocol and registration

This systematic review including the meta-analysis was performed based on the PRISMA statement (The Preferred Reporting Items for Systematic reviews and Meta-Analyses). Also, its protocol was recorded on the PROSPERO registration platform with the registration number CRD42022360353. Ethical approval was not required for this review.

### Focused question

A PICO strategy was defined to establish the focused question: In vitro evaluation of implant–abutment interface (Population), from baseline to end of follow-up, what is the incidence of microleakage events (Outcome) after dynamic loading (Intervention), and what are the key factors (Comparison) that affect sealing ability along the IAI?

### Eligibility criteria

#### Inclusion criteria

1. In vitro studies describing the implant–abutment connection and its resistance to microleakage.
2. Studies investigated microleakage at the IAI with at least 10 samples per group, and each sample consisted of a single implant–abutment connection with or without restoration.
3. If there were multiple publications on the same samples, only the latest one was included.

#### Exclusion criteria

1. Insufficient information about the measurement method, subject numbers, number of leakage samples, measurement timeline, and IAI design.
2. Studies, which only provided bacterial leakage in unloaded or static conditions.
3. Studies provided data on splinted crowns.
4. Clinical studies or in vivo studies.

### Search strategy

The electronic search was conducted in MEDLINE (PubMed), Web of Science, and EMBASE up to September 2022 using a combination of text words and MeSH terms (see Appendix 1). In addition, reference lists of included studies were screened to find potential articles. A manual search of the following journals was also conducted: Journal of Prosthodontics; Clinical Oral Implants Research; Journal of Periodontology; Clinical Implant Dentistry and Related Research; International Journal of Oral & Maxillofacial Implants; Journal of Periodontics and Restorative Dentistry.

### Quality assessment

To assess the quality of eligible studies, 8 items for non-comparative studies and 12 items for comparative studies were evaluated by two reviewers (ZM, QYZ) by using the modified Methodological Index for Non-randomized Studies (MINORS) score [19]. Each item was scored 0 (not reported), 1 (reported but inadequate), or 2 (reported and adequate). The ideal score of each study is 16 for non-comparative studies and 24 for comparative studies. Any discrepancies between the two reviewers were resolved by discussion and inter-examiner agreement was assessed using the kappa coefficient.

### Data collection

Following automatically discarding duplicates, two authors (ZM, DMW) independently screened titles and abstracts of qualified studies. If insufficient information was provided by pertinent abstracts, the full text of articles was required. After the full-text assessment process was finished, ZM and DMW extracted the data independently from the included studies using Microsoft Excel software (Microsoft Office Professional Plus 2016, CA, USA). Any disagreement or accuracy of extracted data between the two reviewers was resolved by discussion with another author (FS).

### Data analysis

All statistical analyses were processed via STATA software (Version 15.1 SE, Stata Corp). The number of implants of each study exhibiting bacterial leakage colonization in the microgap and total sample size were extracted. By definition, the incidence of microleakage events (IME) of each study was calculated by dividing the number of events (the microbial leakage occurring) in the numerator by the total sample size. Data would be extracted as an independent group dataset when the study had multiple qualified groups. The primary outcome of each study was pooled as a weighted mean using a 95% confidence interval (CI) in the random-effect model (DerSimonian–Laird test) due to the high heterogeneity, while the *I*^2^ and *Q*-test were conducted to describe the heterogeneity between studies. Additionally, subgroup analysis and sensitivity analysis were performed to investigate the possible variables causing heterogeneity, and meta-regression analysis was implemented to access the correlation between the outcome and variables. Statistical significance was defined as *p* < 0.05. Publication bias was evaluated using a funnel plot.

## Results

### Literature search and study selection

**Fig. 1:**
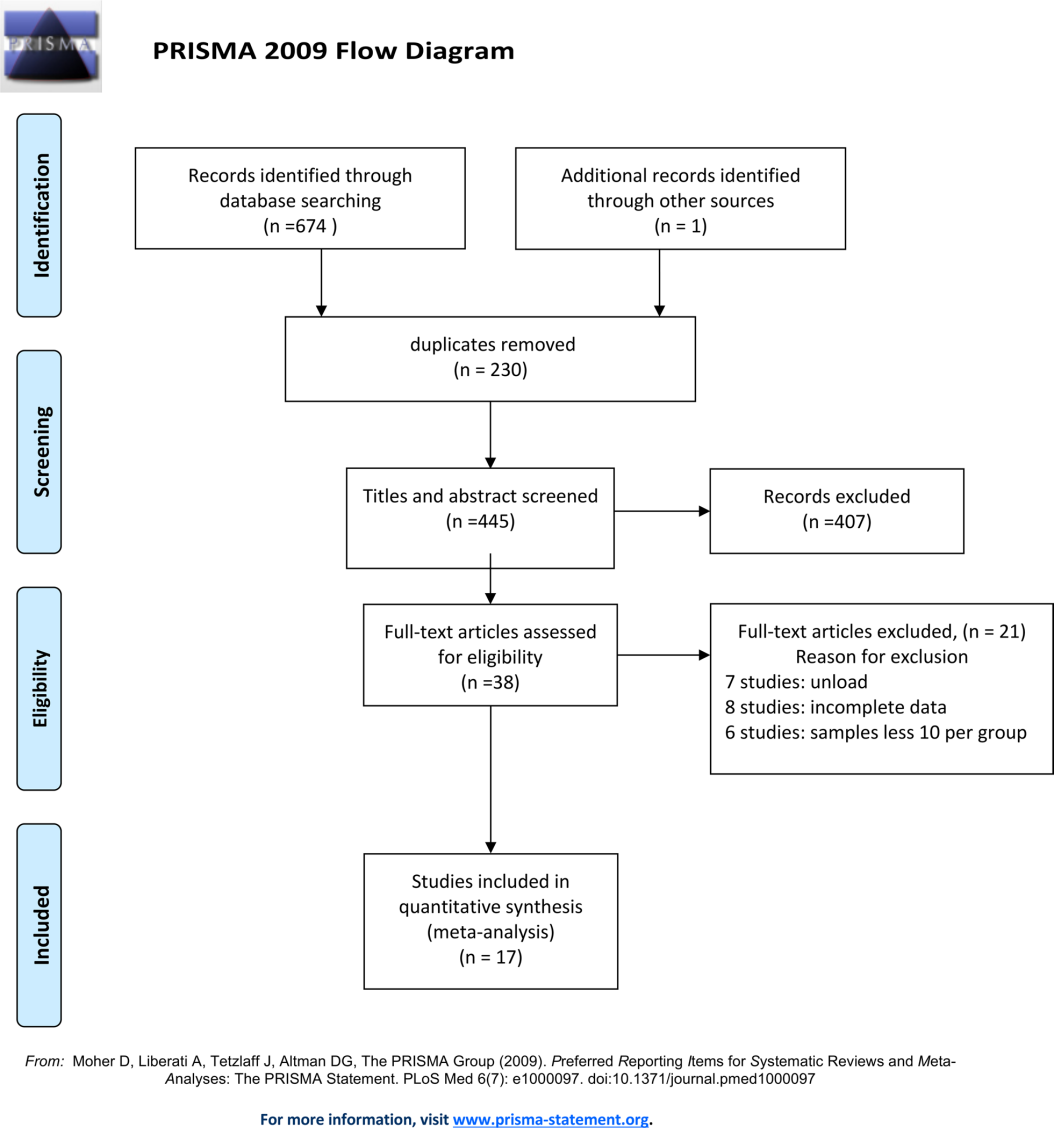
Search strategy

The details of the study selection process are illustrated in Fig. 1. Through the initial search in selected databases and manual search, 675 articles were identified, from which 230 duplicates were excluded. 38 publications were left for full-text reading following titles and abstracts screening. Among these, only 17 studies were considered eligible for qualitative and quantitative synthesis, while others were excluded for different reasons: 7 studies only provided the IME in static situation [13, 14, 20–24]. The sample size of 6 studies did not meet the inclusion criteria [10, 15, 17, 25–27]. 8 publications provided incomplete information of the number of leakage samples [4, 28–34].

### Risk of bias assessment

The quality assessment of each selected study is summarized in Appendix 2. All comparative studies had scores above 16 and one non-comparative study scored 12, which indicated an acceptable quality with a low risk of bias. Of all the items, all selected studies had a clearly stated aim while none of them reported an unbiased assessment of the study endpoint. The kappa coefficients of inter-rater reliability for in vitro studies were 0.919 between the two reviewers (ZM, QYZ).

### Characteristics and methodology of the included studies

Basic information from 17 studies with 45 groups is shown in Tables 1 and 2. A total of 728 implants were included. Of these, 12 studies with 35 groups conducted the “inward method”, three of which used stereomicroscope, scanning electron microscopy, and micro-CT to investigate contamination, respectively [2, 35, 36] and DNA checkerboard technique was reported in one study [7]. In contrast, the “outward method” was reported in 5 studies with 10 groups. For dye solution, the bacterial solution was chosen in 11 studies and among them, *Escherichia coli*, and *Enterococcus faecium* solutions were most widely used. In addition, human saliva, methylene blue, Toluidine blue, and silver nitrate solution were also used as markers in other studies. The volume of the solution inoculated into the inner part of the implants varied from 0.1 to 6.5 μL. Seven studies with 12 groups performed the testing process under static conditions, while dynamic loading was conducted in all studies with 33 groups. Moreover, load cycles varied from 500,000 cycles to 6,000,000 cycles along with various loading forces ranging from 15 to 300 N. The dynamic loading procedures were all applied at the direction of the axis or an angle of 30° ± 2° from the longitudinal axis.

**Table 1:** Features of the included studies

Study	Implant system/number of implants (*n*)	Marker type/volume	Loading parameters/follow-up period	Method of evaluation
1. Zipprich et al. 2016	Ankylos, osseospeed, straumann, Nobel active, Osstem, Bego, Biomet3i, camlog, Xive splus, blueSKY/*n* = 70	*Streptococcus sanguinis, Streptococcus mutans, Actinomyces viscosus, Fusobacterium nucleatum, Veillonella parvula*	0–200 N, 1,200,000 cycles, 30° angle	Inward
2. Koutouzis et al. 2016	Astra, osseospeed/*n* = 40	*Escherichia coli* DH5α	160 N, 500,000 cycles, 1 Hz, 30° angle	Inward
3. Koutouzis et al. 2014	Implant One Fixtures/*n* = 40	*Escherichia coli* DH5α	Unloaded group: 5 days loaded group: 50 N, 500,000 cycles, 1 Hz, 30° angle	Inward
4. Koutouzis et al. 2011	Ankylos, Straumann/*n* = 28	*Escherichia coli* DH5α	15 N, 500,000 cycles, axial loading	Inward
5. Tripodi et al. 2015	Universal II CM, Implacil, De Bortoli, Sao Paulo, Brasils/*n* = 20	*Enterococcus faecalis*, 0.1 μL	120 N, 500,000 cycles, 1 Hz, 90°, 14 days	Outward
6. Ozdiler et al. 2018	Ankylos, bego, Trias, DTI/*n* = 84	*Enterococcus faecium*, 8 mL	50 N, 500,000 cycles, 1 Hz, 30° angle, 4 days	Inward
7. do Nascimento et al. 2012	SIN, Sistema de Implante Nacional/*n* = 60	human saliva (200 μL for unloaded/500 μL for loaded)	Unloaded group: 7 days loaded group: 120 N, 5,000,000 cycles, 1.8 Hz, axial loading	Inward
8. Wachtel et al. 2019	Nobel active/*n* = 10	*Enterococcus faecium*, 6.5 μL	50 N, 1,200,000 cycles, 2 Hz, 30° angle	Outward
9. Ortega-Martínez et al. 2022	MIS Implants Technologies Ltd/*n* = 48	2% methylene blue solution	14 N–160 N, 1,200,000 cycles, 15 Hz, 30° angle	Inward
10. He et al. 2019	Mozo Grau, Spain/*n* = 20	Silver nitrate solution, 1 mL	1000 cycles for each load level, load level: 50, 70, 90, and 100 N for group 1 and 20, 40, 60, and 80 N for group 2. 1 Hz, 30° angle	Inward
11. Amjadi et al. 2021	Tapered Screw‑Vent, Zimmer Dental/*n* = 20	*Escherichia coli*	Unloaded: 5 days, loaded group: 120 N, 500,000 cycles, 1 Hz	Inward
12. Pautke et al. 2009	IMZ, twin plus dentsply/*n* = 60	*Escherichia coli*, 3 μL	50–500 N, 5 Hz, 1,000,000 cycles,	Outward
13. Li et al. 2019	Nobel Replace CC, Straumann, Wego/*n* = 30	Toluidine blue, 3 μL	20–200N, 2 Hz, 48 h	Outward
14. Alves et al. 2016	/*n* = 48	*Escherichia coli*, 75 mL	120 N, 500,000 cycles, 2 Hz, 30° angle	Inward
15. Scarano et al. 2015	Universal II HI and CM, Implacil, De Bortoli, Sao Paulo, Brasil/*n* = 60	Toluidine blue, 0.7 μL	20–300 N, 6,000,000 cycles, 4 Hz, 30° angle	Outward
16. Larrucea Verdugo et al. 2014	MG Mozo-Grau Osseous, MG Mozo-Grau InHex/*n* = 42	0.2% Methylene blue	Occlusal load cycles of axial direction to the implant of 2000 cycles of 10k every 0.5 s	Inward
17. Ellakany et al. 2021	Ankylos/*n* = 48	*Enterococcus faecalis*, S*taphylococcus aureus*, *Pseudomonas aeruginosa*, 200 μL	120 N, 5,000,000 cycles, 2 Hz, axial load, 7 days	Inward

**Table 2:** Influencing factors of the included studies

Study	Group	Sample number	Leakage number	Load or unload	Type of implant connection	Final torque	Abutment material	Sealing agent
1. Zipprich et al. 2016	1	35	1	Dynamic loading	Conical	According to manufacturers’ recommendation	Titanium	–
	2	35	6	Dynamic loading	Flat (internal)	According to manufacturers’ recommendation	Titanium	–
2. Koutouzis et al. 2016	1	20	10	Dynamic loading	Morse taper with conventional marginal design	25 Ncm	Titanium	–
	2	20	8	Dynamic loading	Morse taper with sloped marginal design	25 Ncm	Titanium	–
3. Koutouzis et al. 2014	1	20	1	Unloaded	Morse taper	25 Ncm	Titanium	–
	2	20	4	Dynamic loading	Morse taper	25 Ncm	Titanium	–
4. Koutouzis et al. 2011	1	14	1	Dynamic loading	Morse taper	25 Ncm	Titanium	–
	2	14	12	Dynamic loading	Four-groove conical internal connection	35 Ncm	Titanium	–
5. Tripodi et al. 2015	1	10	2	Dynamic loading	Cone Morse taper	30 Ncm	Titanium	–
	2	10	2	Unloaded	Cone Morse taper	30 Ncm	Titanium	–
6. Ozdiler et al. 2018	1	28	19	Dynamic loading	Internal conical connection	According to manufacturers’ recommendation	Titanium	–
	2	28	7	Dynamic loading	Internal conical connection	According to manufacturers’ recommendation	Titanium	2% chlorhexidine digluconate
	3	28	7	Dynamic loading	Internal conical connection	According to manufacturers’ recommendation	Titanium	Kiero seal (polyvinyl siloxane-based material)
7. do Nascimento et al. 2012	1	10	10	Dynamic loading	External-hexagon	20 Ncm	Titanium	–
	2	10	10	Dynamic loading	Internal-hexagon	20 Ncm	Titanium	–
	3	10	9	Dynamic loading	Morse cone	20 Ncm	Titanium	–
	4	10	3	Unloaded	External-hexagon	20 Ncm	Titanium	–
	5	10	4	Unloaded	Internal-hexagon	20 Ncm	Titanium	–
	6	10	1	Unloaded	Morse cone	20 Ncm	Titanium	–
8. Wachtel et al. 2019	–	10	0	Dynamic loading	Conical connection	15 Ncm	PEEK	–
9. Ortega-Martínez et al. 2022	1	12	7	Unloaded	Internal hexagonal connection	25Ncm	PEEK	–
	2	12	12	Dynamic loading	Internal hexagonal connection	25 Ncm	PEEK	–
	3	12	0	Unloaded	Internal hexagonal connection	25 Ncm	Titanium	–
	4	12	2	Dynamic loading	Internal hexagonal connection	25 Ncm	Titanium	–
10. He et al. 2019	1	10	10	Dynamic loading	Conical connection (11° taper)	20 Ncm	Titanium	–
	2	10	10	Dynamic loading	External hexagonal connection (flat-to-flat)	20 Ncm	Titanium	–
11. Amjadi et al. 2021	1	10	1	Unloaded	Internal connection (slip joint interface)	35 Ncm	Titanium	–
	2	10	5	Dynamic loading	Internal connection (slip joint interface)	35 Ncm	Titanium	–
12. Pautke et al. 2009	1	30	7	Dynamic loading	Internal	Unclear	Titanium	–
	2	30	1	Dynamic loading	Internal	Unclear	Titanium	–
13. Li et al. 2019	1	10	10	Dynamic loading	Morse 6°	35 Ncm	Titanium	–
	2	10	10	Dynamic loading	15° conical	35 Ncm	Titanium	–
	3	10	10	Dynamic loading	Morse 5.75°	20 Ncm	Titanium	–
14. Alves et al. 2016	1	12	1	Unloaded	Conical screwless connection (Morse taper)	–	Titanium	–
	2	12	3	Dynamic loading	Conical screwless connection (Morse taper)	–	Titanium	–
	3	12	7	Unloaded	Tapered screw-retained connection	20 Ncm	Titanium	–
	4	12	5	Dynamic loading	Tapered screw-retained connection	20 Ncm	Titanium	–
15. Scarano et al. 2015	1	30	10	Dynamic loading	external hexagonal connection	Unclear	Titanium	–
	2	30	1	Dynamic loading	Cone Morse taper	–	Titanium	–
16. Larrucea Verdugo et al. 2014	1	21	18	Dynamic loading	Morse taper	Manual, 20 Ncm, 30 Ncm	Titanium	–
	2	21	21	Dynamic loading	External connection	Manual, 20 Ncm, 30 Ncm	Titanium	–
17. Ellakany et al. 2021	1	12	0	Unloaded	Morse taper	15 Ncm	Titanium	–
	2	12	0	Thermocycling	Morse taper	15 Ncm	Titanium	–
	3	12	12	Dynamic loading	Morse taper	15 Ncm	Titanium	–
	4	12	12	Dynamic loading Thermocycling	Morse taper	15 Ncm	Titanium	–

### Implant–abutment connection design

Differences in the implant–abutment connection type for bacterial sealing ability were compared. Internal connections were investigated in 13 studies with 40 groups. Of these, conical connections especially for Morse taper design are mainly used. Conversely, external connections were only evaluated in 4 studies with 5 groups.

### Applied torque value

Almost all included studies followed the manufacturer’s recommendation to apply the closing torque on the abutment components varying from 15 to 35 N, while Verdugo et al. investigated the effect of different final torque on microleakage [2].

### Abutment material

Titanium abutments were used in most publications except for two studies evaluating the sealing ability of PEEK abutments [6, 35]. Unfortunately, no zirconia abutments were included in the present study.

### Sealing agents

Ozdiler et al. [9] compared the effect of antimicrobial agents and silicone-based sealant material on bacterial leakage, whereas no sealing agent was used in the other studies.

### Incidence of microleakage events

**Fig. 2:**
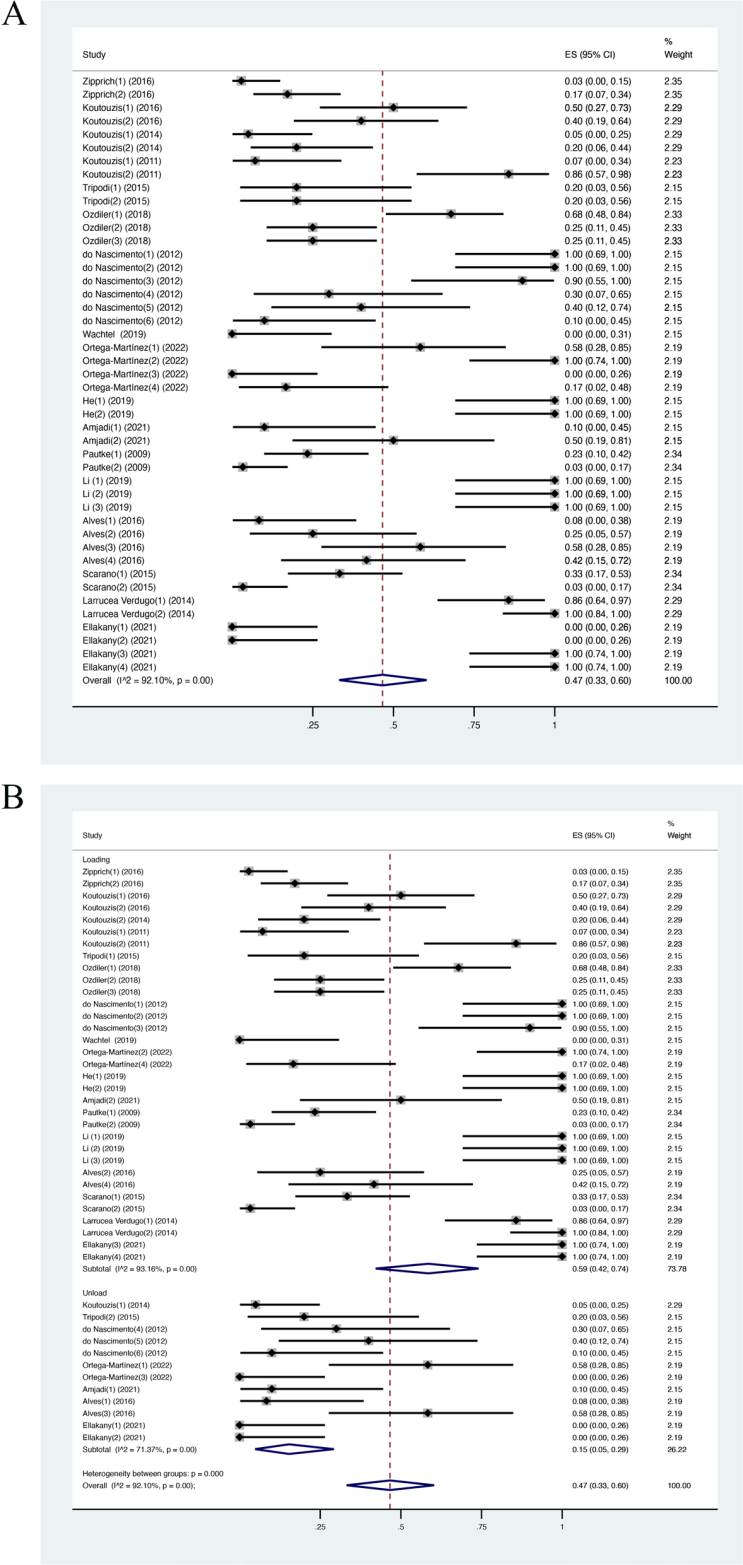
**A** IME in meta-analysis; **B** IME in the subgroup analysis (loaded/unloaded)

In 17 studies, including 45 groups in total, the mean incidence of microleakage events (weighted mean of IME) was 0.47 (CI [0.33, 0.60]; *I*^2^ = 92.10%) (Fig. 2A). The result demonstrated that nearly half of the samples showed microleakage during the test.

### Subgroup analysis and sensitivity analysis

**Fig. 3:**
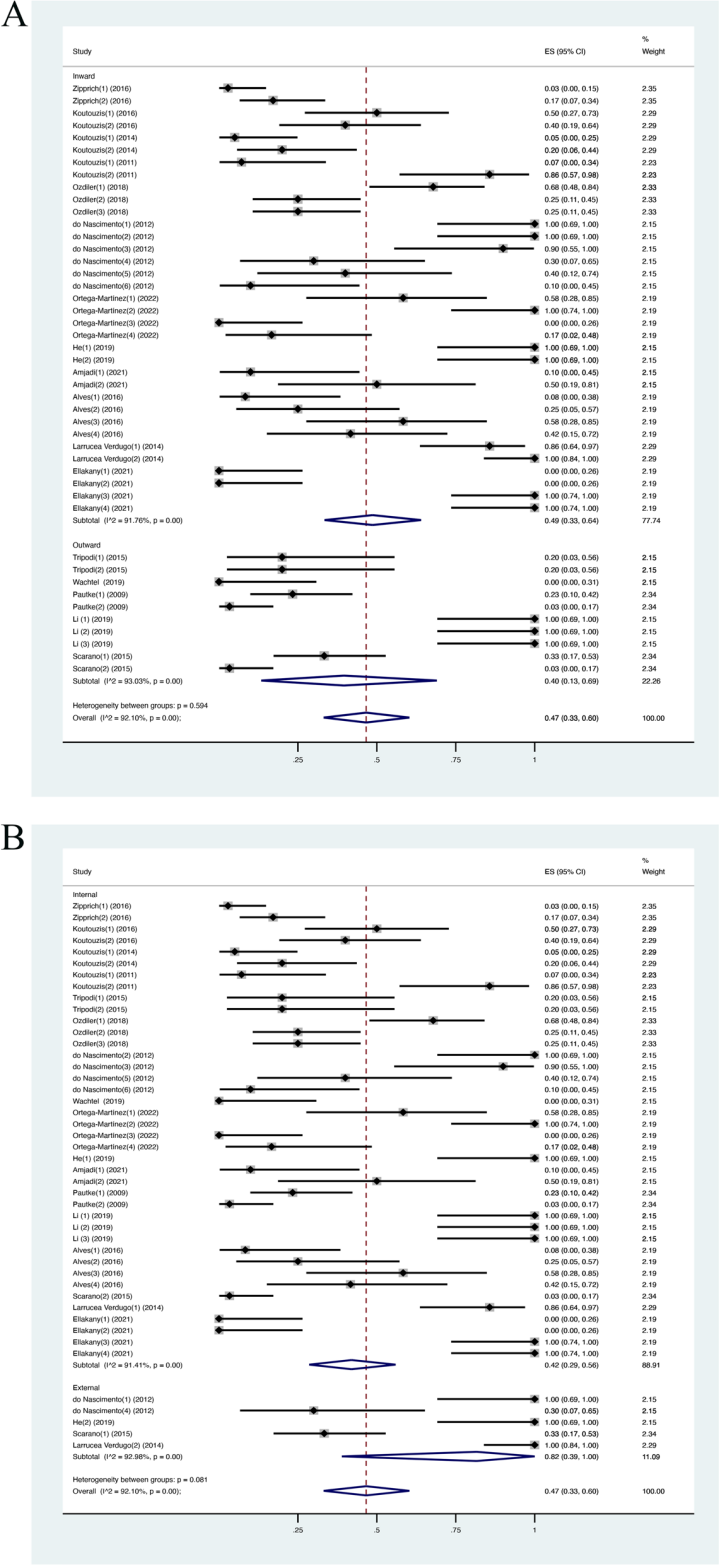
IME in the subgroup analysis (**A** inward/outward; **B** internal/external connection)

**Fig. 4:**
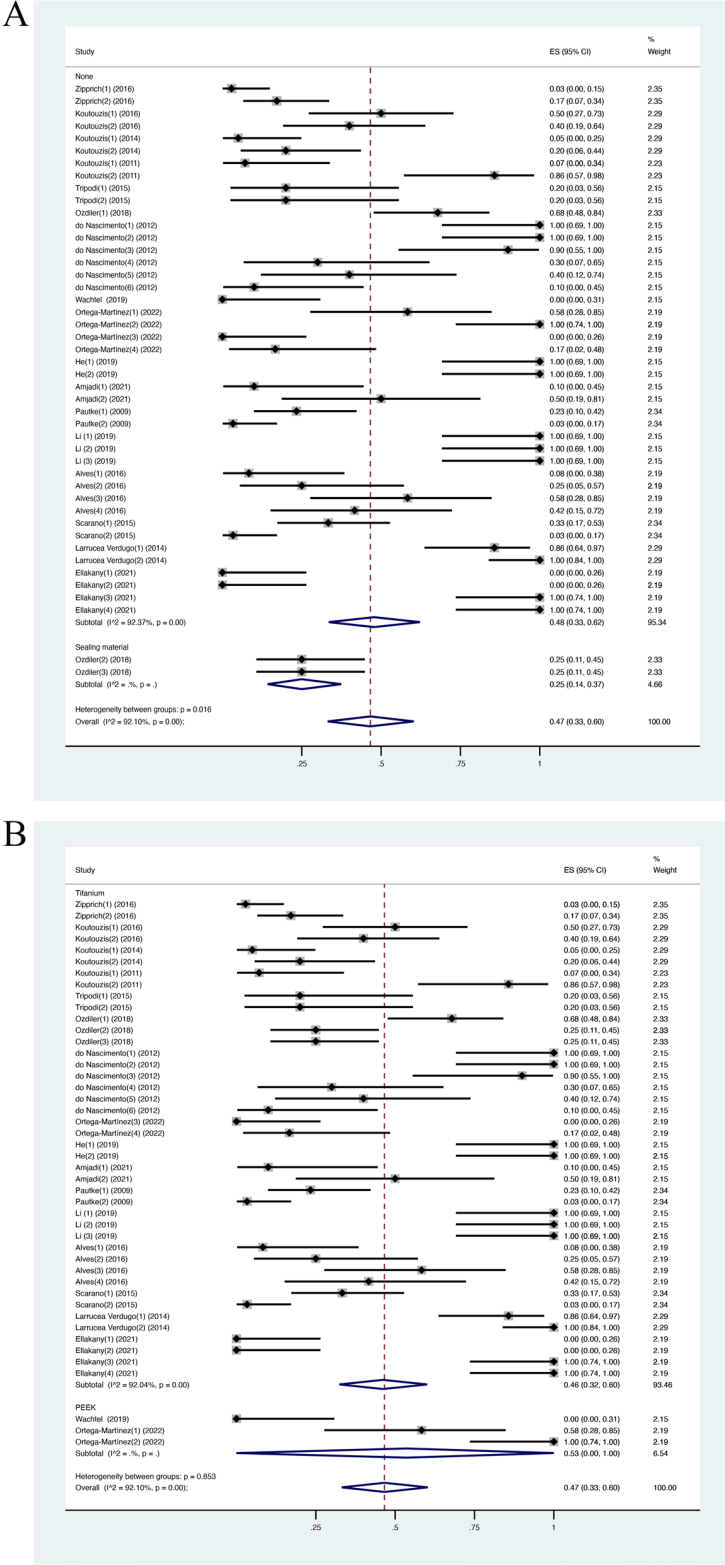
IME in the subgroup analysis (**A** with/without sealing agents placed; **B** titanium/PEEK abutments)

To explain the heterogeneity of estimated microleakage incidence in the included studies, various subgroups were chosen for further analysis: dynamic loading/static condition, inward method/outward method, internal connections/external connections, the use of sealing agent/no sealing agent, titanium abutment/PEEK abutment. The weighted mean IME value was higher in the dynamic loading group, at 0.59 (CI [0.42, 0.74]; *I*^2^ = 93.16%) when compared with the unloaded group, at 0.15 (CI [0.05, 0.29]; *I*^2^ = 71.37%) and heterogeneity between these two groups was significant (Fig. 2B). For the inward method group, the weighted mean IME was 0.49 (CI [0.33, 0.64]; *I*^2^ = 91.76%), while the weighted mean IME of the outward method group was 0.40 (CI [0.13, 0.69]; *I*^2^ = 93.03%) (Fig. 3A). The weighted mean IME in the internal connection group was 0.42 (CI [0.29, 0.56]; *I*^2^ = 91.41%) and 0.82 (CI [0.39, 1]; *I*^2^ = 92.98%) in the external connection group (Fig. 3B). The weighted mean IME of the group without using sealing material was higher, at 0.48 (CI [0.33, 0.62]; *I*^2^ = 92.37%) while the sealing agent group was only at 0.25 (CI [0.14, 0.37]) (Fig. 4A). For abutment type, the weight mean IME of the titanium group and PEEK group was similar, at 0.46 (CI [0.32, 0.60]; *I*^2^ = 92.04%) and 0.53 (CI [0.00, 1]) (Fig. 4B). This demonstrated that albeit most groups were significantly heterogeneous, the dynamic loading process exerted a significant influence on the microleakage along IAI. However, *I*^2^ in the sealing group and PEEK group could not be calculated due to the small sample size so the results from these two groups should be considered carefully. Sensitivity analysis was conducted to evaluate the robustness of the results by omitting each dataset in turn. The highest weighted mean IME was 0.48 (CI [0.36, 0.61]) when Ortega-Martinez [35] was excluded, whereas the lowest weighted mean IME was 0.45 (CI [0.33, 0.57]) when Verdugo [2] was excluded. According to the sensitivity analysis’s results, the results of the present study were stable and not determined by any group or study.

### Meta-regression analysis

To evaluate the correlation of IME with different variables, a meta-regression analysis was conducted. The definitions of these variables and results are shown in Table 3. Among all, the loading condition was the only variable that significantly correlated with IME (*p*-value = 0.016), in other words, dynamic loading significantly displayed more microleakage events when compared with the unloaded group. Likewise, connection design was marginally associated with the IME (*p*-value = 0.091). Other variables were not associated with IME. For variables like final torque value, a meta-regression analysis could not be performed due to the lack of datasets.

**Table 3:** Evaluation of the impact of variables on microleakage incidence using meta-regression analysis

Independent variables	Coefficient**	95% CI*	*P*-value
**Load**	**0.315**	**[0.06; 0.57]**	**0.016**
Approach	− 0.106	[− 0.38; 0.17]	0.436
Connection	0.302	[− 0.05; 0.65]	0.091
Sealing material	− 0.200	[− 0.69; 0.29]	0.415
Abutment material	− 0.112	[− 0.37; 0.60]	0.642

Lines with bold text signify indipendent variables with significant correlation with IME

*95% CI: 95% confidence interval of the coefficient

**Coefficient: a positive value represents a higher incidence of microleakage at the implant–abutment interface in dynamic loading conditions, and vice versa. All factors are binary variables in the analysis. The results mean the loading group had a 31.5% higher incidence than the unloaded group

Approach: inward method versus outward method

Connection: internal connection versus external connection

Sealing material: using sealing material or not

Abutment material: titanium abutments versus PEEK abutments

### Publication bias

No apparent asymmetry distribution was shown in the funnel plot (Appendix 3).

## Discussion

### Incidence of microleakage events

The present review focused on the incidence of microleakage events (IME) at the implant–abutment interface (IAI). The result indicated that 47% of all samples exhibited contamination. This is in accordance with the result of an in vitro study with 45% IME [37]. It should be noted that samples under the dynamic loading test presented higher contamination (IME = 59%), while the data of unloaded samples were lowest, at 15%. This is consistent with a previous study that reported bacterial penetration significantly increased following cyclic loading [16]. In terms of connection designs, internal connection (IME = 42%) appeared to be more resistant to leakage than external connection (IME = 82%), which was supported by other previous in vitro investigations [36, 38].

### The influence of the methodologic aspect on microleakage

#### Loading versus non-loading condition

Applying occlusal force in the test is indispensable since it simulates the masticatory function in the oral cavity. Nascimento et al. [7] demonstrated that dynamic loading may contribute to micromovement of the implant components, resulting in an enlarged microgap in the implant–abutment junction and therefore increased bacterial colonization. This idea was partly supported by the results of the meta-regression analysis in the present review. Consequently, the authors suggest that dynamic loading should be considered as a standard condition in future in vitro microleakage evaluation. However, it is important to point out that the lack of standard criteria of the parameters (e.g., number of loading cycles, loading force, frequency) in the loading process might be a probable reason for heterogeneity between different studies. Meanwhile, Steinebrunner et al. [26] argued that the number of load cycles, until bacterial leakage happened, was dependent on the used implant system. Ozdiler [9] also suggested that higher-level forces and cycles should be conducted in further studies. Moreover, although axis load was reported in several studies, an angle of 30° ± 2° between the longitudinal axis and vertical direction was recommended in ISO standards for dental implants (EN ISO 14801:2016). Thus, the influence of different loading direction on leakage outcomes was also unclear. Generally, due to the lack of consistency among in vitro studies, it would be important to investigate the effect of different parameters of the dynamic loading process on microleakage in future studies.

#### “Inward method” versus “outward method”

Meta-regression analysis did not establish a significant difference between inward (IME = 49%) and outward (IME = 40%) groups, which indicated that these methods are both effective and acceptable. Lack of standardization during decontamination of the surface of implants and abutments, being incapable of showing the exact moment of leakage, confounding factors during abutment removal and maker collecting processes may all lead to false positive results for the “inward method”. On the other hand, the results from the “outward method” were also questionable since it is difficult to ensure whether the dye solution remains in place or bacteria remain active after abutment placement. As a matter of fact, the influence of maker type and volume still remains controversial. It seems that too little solution is adverse to bacterial survival while too much of it may spill out following abutment placement. In summary, contradictory results may be partly explained by different and unreproducible details between in vitro studies and the importance is to standardize all these small influencing variables in further assessment.

### The influence of product aspect on microleakage

#### Implant connections

The design of the IAI is either an internal or external connection, which may be further subclassified according to multiple configurations, such as hexagon, octagon, or conical connection. External hexagon connection was the first and most common connection design in implantology despite several disadvantages, such as great tension in the screw, rotational freedom between platform and restoration component, and little contact sliding between implant head and abutment [1]. In contrast, the internal connection was developed to improve the stability and stress distribution by increasing contact length and passing the screw into the implant body [2]. Verdugo et al. [2] demonstrated that internal connections performed better than external connections in regard to bacterial sealing. This view is similar to the results of the meta-regression analysis in the present study, in which the IME of the internal connection sample is around 30% less than that of external samples. Duyck et al. [39] reported that the average microgap of the hexagonal implant–abutment junction is over 10 µm, while a misfit of 2–3 µm was determined in several studies for internal conical connection implants [20, 40]. A possible explanation is that the unique internal joint design in conical implants provides intimate implant–abutment contact and significant friction locking, which leads to smaller misfits and reduces microbial penetration. Tsuruta et al. [29] indicated that there is a significant difference in the amount of microleakage events between conical connection and parallel connection with an increasing number of loading cycles. Schmitt et al. [3] also revealed that implants utilizing conical connections were superior in sealing performance to the non-conical systems. Moreover, the effect of different taper angles in conical design implants on bacterial contamination remains controversial. Ozdiler et al. [9] examined various conical implants (5.4, 12, 45, and 60 degrees) under loaded conditions and revealed no significant difference in the frequency of bacterial leakage with different taper angles.

#### Abutment material

Sen et al. [20] demonstrated that titanium external abutments were less resistant to bacterial leakage than zirconia external samples under unloaded conditions. In contrast, Smith et al. [41] reported that zirconia abutments showed the largest microgap at 26.7 µm, whereas they found the microgap in titanium abutments to be only 2 µm. Also, the rougher surface of zirconia abutments may induce more adhesion of microorganisms. Furthermore, Wachtel et al. [6] assessed 10 polyetheretherketone (PEEK) abutment–crown–complex connections with conical design under dynamic loading conditions and no contamination occurred during the whole follow-up period. Compared with high rigidity materials like titanium and zirconia with an elastic modulus of 110 GPa and 210 GPa, respectively, PEEK, as an elastic material with a comparatively low elastic modulus of 3.5 GPa might be an ideal abutment material to prevent micromovements along the IAI [42, 43]. However, Martínez et al. [35] suggested that bacterial tightness and mechanical properties were better in titanium groups compared with PEEK material. Due to the limited data of the included studies, sealing ability in different materials is still unclear. Further well-conducted in vitro studies with homogeneity are required.

#### The use of sealing agents

Ozdiler et al. [9] demonstrated that the use of silicone sealant or 2% chlorhexidine gel reduced the bacterial leakage counts. Similarly, Besimo et al. [44] observed no contamination at the IAI in all samples when chlorhexidine was applied for 11 weeks of follow-up. On the other hand, Yu et al. [31] reported that sealing gel decreased the microleakage of the Straumann implant system while no significant difference was found for the Nobel system. In contrast, Duarte et al. [45] found that sealing varnish was incapable of eliminating bacterial penetration. No significant correlation was found in microleakage with the use of sealing material in the present study. Since the number of samples with sealing agent placement was limited, the results should be interpreted carefully. The necessity of sealing gel as well as disinfectant placed at the implant–abutment surface is inconclusive.

#### Applied torque

Most included studies applied final torque following the manufacturer’s recommendation. Larrucea et al. [10] observed internal conical implants with different final torque applied (< 10 N, 10 N, 20 N, 30 N), and contamination only occurred in < 10 and 10 N groups. Several studies [2, 46] also suggested that microleakage decreases when higher torque is used. Since the number of included implants that did not meet the manufacturer’s recommendation was extremely low, quantitative analysis could not be conducted. The influence of final torque on microleakage should be investigated in more in vitro studies.

### Others

It should be noted that several observation methodologies or potential factors could not be assessed in the present study due to the lack of data, such as the use of scanning electron microscopy, X-ray radiography techniques, thermocycling conditions, and the effect of the follow-up period on bacterial penetration between studies.

## Conclusion

Within the limitations of this study, it can be concluded that the dynamic loading process could contribute to a higher incidence of microleakage at the implant–abutment junction, which should be considered a standard step in future for in vitro microleakage evaluation. More well-conducted trials with homogeneous methodologies need to be performed to standardize the in vitro microleakage assessment process.

## Data Availability

The datasets used and analyzed during the current study are available from the corresponding author on reasonable request.

## Appendix 1

PubMed:

Search: ((((((((((((dental implants[MeSH Terms]) OR (Implant, Dental[Text Word])) OR (Implants, Dental[Text Word])) OR (Dental Implant[Text Word])) OR (Dental Prostheses, Surgical[Text Word])) OR (Dental Prosthesis, Surgical[Text Word])) OR (Surgical Dental Prostheses[Text Word])) OR (Surgical Dental Prosthesis[Text Word])) OR (Prostheses, Surgical Dental[Text Word])) OR (Prosthesis, Surgical Dental[Text Word])) OR (((((((((((((Dental Implantation[MeSH Terms]) OR (Dental Implant Therapy[Text Word])) OR (Dental Implant Therapies[Text Word])) OR (Implant Therapy, Dental[Text Word])) OR (Therapy, Dental Implant[Text Word])) OR (Prosthesis Implantation, Dental[Text Word])) OR (Dental Prosthesis Implantation[Text Word])) OR (Implantation, Dental[Text Word])) OR (Implantation, Dental Prosthesis[Text Word])) OR (Dental Prosthesis Implantations[Text Word])) OR (implant dentistry[Text Word])) OR (endosseal implant[Text Word])) OR (dental implantology[Text Word]))) AND (((((((((((((((((((((((((((((((((((((((((((Dental Implant-Abutment Design[MeSH Terms]) OR (Dental Implant Abutment Design[Text Word])) OR (Design, Dental Implant-Abutment[Text Word])) OR (Designs, Dental Implant-Abutment[Text Word])) OR (Implant-Abutment Design, Dental[Text Word])) OR (Implant-Abutment Designs, Dental[Text Word])) OR (Dental Implant-Abutment Designs[Text Word])) OR (Dental Implant Abutment Designs[Text Word])) OR (Dental Implant-Abutment Interface[Text Word])) OR (Dental Implant Abutment Interface[Text Word])) OR (Dental Implant Abutment Interface[Text Word])) OR (Implant-Abutment Interface, Dental[Text Word])) OR (Implant-Abutment Interfaces, Dental[Text Word])) OR (Interface, Dental Implant-Abutment[Text Word])) OR (Interfaces, Dental Implant-Abutment[Text Word])) OR (Dental Implant-Abutment Connection[Text Word])) OR (Connection, Dental Implant-Abutment[Text Word])) OR (Connections, Dental Implant-Abutment[Text Word])) OR (Dental Implant Abutment Connection[Text Word])) OR (Dental Implant-Abutment Connections[Text Word])) OR (Implant-Abutment Connection, Dental[Text Word])) OR (Implant-Abutment Connections, Dental[Text Word])) OR (Morse Taper Dental Implant-Abutment Interface[Text Word])) OR (Morse Taper Dental Implant Abutment Interface[Text Word])) OR (Morse Taper Dental Implant-Abutment Connection[Text Word])) OR (Morse Taper Dental Implant Abutment Connection[Text Word])) OR (Dental Implant Platform Switching[Text Word])) OR (Platform Switching, Dental Implant[Text Word])) OR (fixture-abutment interface[Text Word])) OR (fixture–abutment junction[Text Word])) OR (Implant Abutment Interface (IAI[Text Word]))) OR (Implant-abutment junction[Text Word])) OR (Cone Morse connections[Text Word])) OR (Cone Morse implant-abutment connection[Text Word])) OR (flat-to-flat connections,[Text Word])) OR (tube-in-tube connections[Text Word])) OR (external hexagon[Text Word])) OR (internal hexagon[Text Word])) OR (implant platforms[Text Word])) OR (two pieces dental implants[Text Word])) OR (internal Morse-taper connection[Text Word])) OR (four-groove conical internal connection[Text Word])) OR (internal conical implant systems[Text Word]))) AND (((((((((((((((((((((((((((((((((((dental leakage[MeSH Terms]) OR (Leakages, Dental[Text Word])) OR (Dental Leakages[Text Word])) OR (Leakage, Dental[Text Word])) OR (bacterial microleakage[Text Word])) OR (bacterial reservoir[Text Word])) OR (bacterial contamination[Text Word])) OR (bacterial leakage[Text Word])) OR (Bacterial Colonization[Text Word])) OR (bacterial aggregation[Text Word])) OR (bacterial invasion[Text Word])) OR (bacterial penetration[Text Word])) OR (bacterial infiltrate[Text Word])) OR (Bacterial migration[Text Word])) OR (bacterial microfiltration[Text Word])) OR (microbial microleakage[Text Word])) OR (microbial reservoir[Text Word])) OR (microbial contamination[Text Word])) OR (microbial leakage[Text Word])) OR (microbial Colonization[Text Word])) OR (microbial aggregation[Text Word])) OR (microbial invasion[Text Word])) OR (microbial penetration[Text Word])) OR (microbial infiltrate[Text Word])) OR (microbial migration[Text Word])) OR (microbial microfiltration[Text Word])) OR (microbiologic[Text Word])) OR (Bacterial biofilm accumulation[Text Word])) OR (oral micro-organisms[Text Word])) OR (microleakage[Text Word])) OR (oral microorganisms[Text Word])) OR (Microbiological Sealing[Text Word])) OR (Bacterial sealing[Text Word])) OR (sealant[Text Word])) OR (sealing[Text Word]))

## EMBASE:

(('dental implants'/exp OR 'implant, dental':ab,ti OR 'implants, dental':ab,ti OR 'dental implant':ab,ti OR 'dental prostheses, surgical':ab,ti OR 'dental prosthesis, surgical':ab,ti OR 'surgical dental prostheses':ab,ti OR 'surgical dental prosthesis':ab,ti OR 'prostheses, surgical dental':ab,ti OR 'prosthesis, surgical dental':ab,ti OR 'dental anchor':ab,ti OR 'endosseous implant':ab,ti OR 'root canal post':ab,ti OR 'single tooth implant':ab,ti OR 'subperiosteal implant':ab,ti OR 'transgingival implant':ab,ti OR 'transmandibular implant':ab,ti) OR ('dental implantation'/exp OR 'dental implant therapy':ab,ti OR 'dental implant therapies':ab,ti OR 'implant therapy, dental':ab,ti OR 'therapy, dental implant':ab,ti OR 'prosthesis implantation, dental':ab,ti OR 'dental prosthesis implantation':ab,ti OR 'implantation, dental':ab,ti OR 'implantation, dental prosthesis':ab,ti OR 'dental prosthesis implantations':ab,ti OR 'implant dentistry':ab,ti OR 'endosseal implant':ab,ti OR 'dental implantology':ab,ti) OR ('dental implantation'/exp OR 'dental implant therapy':ab,ti OR 'dental implant therapies':ab,ti OR 'implant therapy, dental':ab,ti OR 'therapy, dental implant':ab,ti OR 'prosthesis implantation, dental':ab,ti OR 'dental prosthesis implantation':ab,ti OR 'implantation, dental':ab,ti OR 'implantation, dental prosthesis':ab,ti OR 'dental prosthesis implantations':ab,ti OR 'implant dentistry':ab,ti OR 'endosseal implant':ab,ti OR 'dental implantology':ab,ti)) AND ('dental implant-abutment design'/exp OR 'dental implant abutment design':ab,ti OR 'design, dental implant-abutment':ab,ti OR 'designs, dental implant-abutment':ab,ti OR 'implant-abutment design, dental':ab,ti OR 'implant-abutment designs, dental':ab,ti OR 'dental implant-abutment designs':ab,ti OR 'dental implant abutment designs':ab,ti OR 'dental implant-abutment interface':ab,ti OR 'dental implant abutment interface':ab,ti OR 'implant-abutment interface, dental':ab,ti OR 'implant-abutment interfaces, dental':ab,ti OR 'interface, dental implant-abutment':ab,ti OR 'interfaces, dental implant-abutment':ab,ti OR 'dental implant-abutment connection':ab,ti OR 'connection, dental implant-abutment':ab,ti OR 'connections, dental implant-abutment':ab,ti OR 'dental implant abutment connection':ab,ti OR 'dental implant-abutment connections':ab,ti OR 'implant-abutment connection, dental':ab,ti OR 'implant-abutment connections, dental':ab,ti OR 'morse taper dental implant-abutment interface':ab,ti OR 'morse taper dental implant abutment interface':ab,ti OR 'morse taper dental implant-abutment connection':ab,ti OR 'morse taper dental implant abutment connection':ab,ti OR 'dental implant platform switching':ab,ti OR 'platform switching, dental implant':ab,ti OR 'fixture-abutment interface':ab,ti OR 'fixture–abutment junction':ab,ti OR ('implant abutment interface':ab,ti AND iai:ab,ti) OR 'implant-abutment junction':ab,ti OR 'cone morse connections':ab,ti OR 'cone morse implant-abutment connection':ab,ti OR 'flat-to-flat connections,':ab,ti OR 'external hexagon':ab,ti OR 'implant platforms':ab,ti OR 'tube-in-tube connections':ab,ti OR 'internal hexagon':ab,ti OR 'two pieces dental implants':ab,ti OR 'internal morse-taper connection':ab,ti OR 'four-groove conical internal connection':ab,ti OR 'internal conical implant systems':ab,ti AND ('dental leakage':ab,ti OR 'leakages, dental':ab,ti OR 'dental leakages':ab,ti OR 'leakage, dental':ab,ti OR 'bacterial microleakage':ab,ti OR 'bacterial reservoir':ab,ti OR 'bacterial contamination':ab,ti OR 'bacterial leakage':ab,ti OR 'bacterial colonization':ab,ti OR 'bacterial aggregation':ab,ti OR 'bacterial invasion':ab,ti OR 'bacterial penetration':ab,ti OR 'bacterial infiltrate':ab,ti OR 'bacterial migration':ab,ti OR 'bacterial microfiltration':ab,ti OR 'microbial microleakage':ab,ti OR 'microbial reservoir':ab,ti OR 'microbial contamination':ab,ti OR 'microbial leakage':ab,ti OR 'microbial colonization':ab,ti OR 'microbial aggregation':ab,ti OR 'microbial invasion':ab,ti OR 'microbial penetration':ab,ti OR 'microbial infiltrate':ab,ti OR 'microbial migration':ab,ti OR 'microbial microfiltration':ab,ti OR microbiologic:ab,ti OR 'bacterial biofilm accumulation':ab,ti OR 'oral micro-organisms':ab,ti OR microleakage:ab,ti OR 'oral microorganisms':ab,ti OR 'microbiological sealing':ab,ti OR 'bacterial sealing':ab,ti OR sealant:ab,ti OR sealing:ab,ti)

Web of science:

(((((((((((((ental Implantation (Topic))or Dental Implant Therapy (Topic))or Dental Implant Therapies (Topic)) or Implant Therapy, Dental (Topic)) or Therapy, Dental Implant (Topic)) or Prosthesis Implantation, Dental (Topic)) or Dental Prosthesis Implantation (Topic)) or Implantation, Dental (Topic)) or Implantation, Dental Prosthesis (Topic)) or Dental Prosthesis Implantations (Topic)) or implant dentistry (Topic)) or endosseal implant (Topic)) or dental implantology (Topic)) or ((((((((((((((((((dental implants (Topic)) or Implant, Dental (Topic)) or Implants, Dental (Topic)) or Dental Implant (Topic)) or Dental Prostheses, Surgical (Topic)) or Dental Prosthesis, Surgical (Topic)) or Surgical Dental Prostheses (Topic)) or Surgical Dental Prosthesis (Topic)) or Prostheses, Surgical Dental (Topic)) or Prosthesis, Surgical Dental (Topic)) or dental anchor (Topic)) or dental pin (Topic)) or endosseous implant (Topic)) or root canal post (Topic)) or single tooth implant (Topic)) or subperiosteal implant (Topic)) or transgingival implant (Topic)) or transmandibular implant (Topic)) and (((((((((((((((((((((((((((((((((((((((((((Dental Implant-Abutment Design (Topic) and Dental Implant Abutment Design (Topic))or Design, Dental Implant-Abutment (Topic)) or Designs, Dental Implant-Abutment (Topic)) or Implant-Abutment Design, Dental (Topic)) or Implant-Abutment Designs, Dental (Topic)) or Dental Implant-Abutment Designs (Topic)) or Dental Implant Abutment Designs (Topic)) or Dental Implant-Abutment Interface (Topic)) or Dental Implant Abutment Interface (Topic)) or Dental Implant Abutment Interface (Topic)) or Implant-Abutment Interface, Dental (Topic)) or Implant-Abutment Interfaces, Dental (Topic)) or Interface, Dental Implant-Abutment (Topic)) or Interfaces, Dental Implant-Abutment (Topic)) or Dental Implant-Abutment Connection (Topic)) or Connection, Dental Implant-Abutment (Topic)) or Connections, Dental Implant-Abutment (Topic)) or Dental Implant Abutment Connection (Topic)) or Dental Implant-Abutment Connections (Topic)) or Implant-Abutment Connection, Dental (Topic)) or Implant-Abutment Connections, Dental (Topic)) or Morse Taper Dental Implant-Abutment Interface (Topic)) or Morse Taper Dental Implant Abutment Interface (Topic)) or Morse Taper Dental Implant-Abutment Connection (Topic)) or Morse Taper Dental Implant Abutment Connection (Topic)) or Dental Implant Platform Switching (Topic)) or Platform Switching, Dental Implant (Topic)) or fixture-abutment interface (Topic)) or fixture–abutment junction (Topic)) or Implant Abutment Interface (IAI) (Topic)) or Implant-abutment junction (Topic)) or Cone Morse connections (Topic)) or Cone Morse implant-abutment connection (Topic)) or flat-to-flat connections, (Topic)) or external hexagon (Topic)) or implant platforms (Topic)) or tube-in-tube connections (Topic)) or internal hexagon (Topic)) or two pieces dental implants (Topic)) or internal Morse-taper connection (Topic)) or four-groove conical internal connection (Topic)) or internal conical implant systems (Topic)) and (((((((((((((((((((((((((((((((((((dental leakage (Topic)) or Leakages, Dental (Topic)) or Dental Leakages (Topic)) or Leakage, Dental (Topic)) or bacterial microleakage (Topic)) or bacterial reservoir (Topic)) or bacterial contamination (Topic)) or bacterial leakage (Topic)) or Bacterial Colonization (Topic)) or bacterial aggregation (Topic)) or bacterial invasion (Topic)) or bacterial penetration (Topic)) or bacterial infiltrate (Topic)) or Bacterial migration (Topic)) or bacterial microfiltration (Topic)) or microbial microleakage (Topic)) or microbial reservoir (Topic)) or microbial contamination (Topic)) or microbial leakage (Topic)) or microbial Colonization (Topic)) or microbial aggregation (Topic)) or microbial invasion (Topic)) or microbial penetration (Topic)) or microbial infiltrate (Topic)) or microbial migration (Topic)) or microbial microfiltration (Topic)) or microbiologic (Topic)) or Bacterial biofilm accumulation (Topic)) or oral micro-organisms (Topic)) or microleakage (Topic)) or oral microorganisms (Topic)) or Microbiological Sealing (Topic)) or Bacterial sealing (Topic)) or sealant (Topic)) or sealing (Topic))

## Appendix 2. The modified Methodological Index for Non-randomized Studies (MINORS) score

**Table:**

Study	A clearly stated aim	Inclusion of consecutive patients	Prospective collection of data	Endpoints appropriate to the aim of the study	Unbiased assessment of the study endpoint:	Follow-up period appropriate to the aim of the study	Loss to follow-up less than 5%	Prospective calculation of the study size
1. Zipprich et al. 2016	2	2	2	2	0	2	2	0
2. Koutouzis et al. 2016	2	2	2	2	0	2	2	0
3. Koutouzis et al. 2014	2	2	2	2	0	2	2	0
4. Koutouzis et al. 2011	2	2	2	2	0	2	2	0
5. Tripodi et al. 2015	2	2	2	2	0	2	2	0
6. Ozdiler et al. 2018	2	2	2	2	0	2	2	2
7. do Nascimento et al. 2012	2	2	2	2	0	2	2	0
8. Wachtel et al. 2019	2	2	2	2	0	2	2	0
9. Ortega-Martínez et al. 2022	2	2	2	2	0	2	2	0
10. He et al. 2019	2	2	2	2	0	1	2	0
11. Amjadi et al. 2021	2	2	2	2	0	2	2	0
12. Pautke et al. 2009	2	2	2	2	0	2	2	0
13. Li et al. 2019	2	2	2	2	0	2	2	0
14. Alves et al. 2016	2	2	2	2	0	2	2	0
15. Scarano et al. 2015	2	2	2	2	0	2	2	0
16. Larrucea Verdugo et al. 2014	2	2	2	1	0	2	2	0
17. Ellakany et al. 2021	2	2	2	2	0	2	2	0

**Table:**

Study	Addition	An adequate control group	Contemporary groups	Baseline equivalence of groups	Adequate statistical analyses	Total
1. Zipprich et al. 2016		2	2	1	2	19
2. Koutouzis et al. 2016		2	2	2	2	20
3. Koutouzis et al. 2014		2	2	2	2	20
4. Koutouzis et al. 2011		2	2	2	2	20
5. Tripodi et al. 2015		2	2	1	1	18
6. Ozdiler et al. 2018		2	2	2	2	22
7. do Nascimento et al. 2012		2	2	2	2	20
8. Wachtel et al. 2019		–	–	–	–	12
9. Ortega-Martínez et al. 2022		2	2	2	2	20
10. He et al. 2019		2	2	2	2	19
11. Amjadi et al. 2021		2	2	2	1	19
12. Pautke et al. 2009		2	2	2	2	20
13. Li et al. 2019		2	2	2	2	20
14. Alves et al. 2016		2	2	1	1	18
15. Scarano et al. 2015		2	2	1	1	18
16. Larrucea Verdugo et al. 2014		2	2	1	2	18
17. Ellakany et al. 2021		2	2	2	1	19

## Abbreviations

IME: The incidence of microleakage events
IAI: Implant–abutment interface
PEEK: Polyetheretherketone
CI: Confidence interval
MINORS: Modified Methodological Index for Non-randomized Studies score
PRISMA: The Preferred Reporting Items for Systematic reviews and Meta-Analyses
